# Dental Caries

**Published:** 1876-05

**Authors:** 


### ARTICLE IX.

### *Dental Caries.*

In a review of a dental work by Mr. Sewell, in the *Med. Press and Circular*, the writer says:

Of all the different subjects, however, with which dentists have to deal, there is none in which the pnblic are so interested, or on which medical men are so frequently asked to pass an opinion, as that of the cause and nature of dental caries. There is a very general impression among the community that dentists cannot satisfactorily account for the havoc which this disease causes among the teeth of nearly every third person we meet with, and for the fact that we seldom find a sound set of teeth in any individual over thirty or forty years of age. But this is a great mistake, as dental pathologists can give as good an explanation of the causes of the deterioration of teeth as can be given in respect of insanity or any other disease which has likewise increased within the last fifty years. The theory of the etiology and pathology of caries which our author has adopted is, to use his own words, "entirely based upon generally admitted facts; it is that which I believe can alone be arrived at by reasoning upon such facts; it is that which has been enunciated by the best authorities, and eventually must be, in my opinion, universally accepted." These causes are to be found in the action of acids, which occasions a gradual softening and disintegration of the dental tissues, the origin and progress of the disease being favored by certain structural defects in the enamel and dentine, and by certain derangements of the general health. "The local and constitutional diseases which favor the onset and progress of caries are those which are accompanied by inflammation of the oral mucous membrane, and those which give rise to the formation or deposit of acid within the mouth. Among the former may be particularly enumerated all the varieties of stomatitis; among the latter scrofula, syphilis, phthisis, diabetes, chlorosis, and chronic alcoholism. These constitutional affections exert their baneful effect upon the teeth, in great part, by reason of the chronic inflammation of the gums, the vitiation (even general acidity) of the secretions of the mouth, and the dyspepsia with which they are all so commonly accompanied." A curious fact in connection with dental caries is that the teeth are not all equally liable to decay; and Mr. Sewill gives the statistics of 10,000 cases collected by Magitot, which show the great relative frequency of caries in the first molars, as well as the much greater frequency of the disease in the front teeth of the upper than in those of the lower jaw. The latter circumstance may, in the opinion of the author, "be accounted for by the fact that the lower front teeth are protected from the action of acid by the saliva with which owing to their position, they are bathed." He admits however, that no entirely satisfactory explanation of the above facts has yet been afforded.—*Med. and Surg. Rep.rter.*

